# Vimentin in Developing Human Adrenal Medulla Cells

**DOI:** 10.3390/life16081295

**Published:** 2026-08-06

**Authors:** Ekaterina Otlyga, Dmitry Otlyga, Yuliya Krivova, Alexandra Proshchina, Olga Junemann, Marina Shahina, Sergey Saveliev

**Affiliations:** 1 Federal State Budgetary Scientific Institution “Petrovsky National Research Centre of Surgery”, 2, Building 1, Abrikosovsky Pereulok, Moscow 119435, Russia; otlyga@bk.ru (D.O.); homulkina@rambler.ru (Y.K.); proshchina@yandex.ru (A.P.); junemann@outlook.com (O.J.); embrains@mail.ru (S.S.); 2 Krasnopolsky Moscow Regional Research Institute of Obstetrics and Gynecology, Pokrovka Street, 22A, Moscow 101000, Russia; shahinamarina@mail.ru

**Keywords:** adrenal medulla, vimentin, tyrosine hydroxylase, human development

## Abstract

The development of the human adrenal gland involves dramatic structural reorganization, including migration of adrenal medulla cells, changes in cell shape, and complex dynamics of distinct cell populations. These processes require dynamic remodeling of the cytoskeleton. In this study, we investigated vimentin—a key regulator of cell shape changes—in a population of developing adrenal medulla cells, which we call large cells (LCs). We performed double immunofluorescence for tyrosine hydroxylase and vimentin on adrenal glands from human fetuses aged from 8 gestational weeks to 11 days postnatally and compared our findings with recently published single-cell transcriptomics data. We found that the ratio of vimentin-positive LCs did not change significantly with age, and staining intensity was even higher in later fetuses, indicating that vimentin immunoreactivity does not decline with age. In contrast, analysis of the scRNA-seq dataset revealed a decrease in the proportion of vimentin-positive cells within the subpopulation of late chromaffin cells. We attribute these differences to both the high stability of vimentin filaments relative to their short-lived mRNA and the physiological heterogeneity of developing chromaffin cells, the precise causes of which remain to be determined.

## 1. Introduction

The ontogeny of the human adrenal medulla (AM) remains a highly underinvestigated problem in developmental biology. In our previous work, we described developmental parallels between the human AM and extra-adrenal chromaffin organs known as the organs of Zuckerkandl (OZs) [1]. We demonstrated that, as organs, the OZs significantly outpace the AM in their development. Thus, while the OZs already represent clearly defined structures, the AM is represented by groups of cells scattered between cortical cells. While the developmental changes in the OZs are mostly limited to changes in the size of the organs themselves and their cells, the changes taking place in the developing AM are dramatic.

To describe these changes, we identified two morphological cell types—large cells (LCs) and small cells (SCs)—which form the developing AM, OZs and ganglia. Firstly, the LCs likely migrate from the OZ anlagen to the adrenal anlage, comprising the primary LC subpopulation in the AM. SCs are undifferentiated cells, migrating to the AM along nerves. After 16 gws (gestational weeks), their number increases rapidly, forming large rounded to oval structures. After 22–23 gws, these SCs likely differentiate into secondary LCs, so that the LC/SC ratio rapidly decreases, and LCs become the predominant cell type. Thus, these primary and secondary LCs correspond to populations of chromaffin cells of varying degrees of maturity, while the SCs likely correspond to so-called Schwann cell precursors and sympathoblasts. Although the massive differentiation of SCs into LCs occurs approximately in the middle of antenatal life, even after this event and after birth, the AM is far from the maturity that is seen in human adults. The AM becomes more circumscribed and better defined, but still, many small groups of SCs are present in different parts of the adrenal gland.

What is more, dramatic changes in the cortical tissue also take place after birth. First of all, the involution of the fetal cortex occurs [2,3] accompanied by massive fetal cortex cell degradation and hyperemia, ending with the formation of a connective tissue layer between the AM and the permanent cortex. This process is fully completed only by the 1st year of life. The so-called permanent cortex also undergoes significant structural changes before birth and postnatally [4]. This reconstruction includes the subdivision of the lobules of the outer cortex, which increases the contact of cortical cells with interstitial capillaries, which was suggested to be the anatomical expression of functional maturation of the adrenal cortex [4]. All these changes are inevitably accompanied by changes in cell shapes. For this, cells require significant reconstruction of the cytoskeleton, and one of the most important conductors of this process is the intermediate filament protein vimentin [5].

In our recent work [1], we showed that with increasing age, βIII-tubulin expression in LCs decreased, suggesting that some other cytoskeletal protein might take the leading role in maintaining or reconstructing cell shape, and a possible candidate for this is vimentin.

Vimentin is also known as a marker of epithelial–mesenchymal transition and migration [6,7]. It is known to be positive in migrating neural crest cells at the onset of their migration to different organs [5,8]. Later, with the differentiation of neural crest cells into neurons, they lose vimentin, which is replaced by neurofilaments [5]. However, such processes are poorly investigated, if at all, in developing chromaffin cells, especially in human embryos and fetuses.

Vimentin is traditionally claimed to be absent in the AM, at least in adult animals, such as guinea pigs [9], rats [10], and cattle [11]. However, this question has some controversies. For instance, Bertelli E. and co-authors found so-called ‘vimentin-like immunoreactivity’ in the perinuclear region of adult rat adrenomedullary cells [12]. According to these authors, the presence of nestin in the AM indirectly proves the presence of vimentin, as nestin has been shown to be unable to form filaments without co-assembling with vimentin. Moreover, S. C. Henzen-Logmans et al. [13] found a positive vimentin reaction in 50% of chromaffin cells in the adult human AM.

Data on vimentin immunoreactivity in human adrenal development are scarce. Therefore, we decided to use double immunofluorescence to detect vimentin in LCs positive for tyrosine hydroxylase (TH+) at various ages during antenatal and early postnatal periods. We focused our vimentin analysis on LCs rather than on SCs. Unlike SCs, LCs lose βIII-tubulin with age, which likely reflects their need for shape changes, a process in which vimentin may be involved. Moreover, LCs are more differentiated than SCs and represent the cell population that will later form the adrenal medulla.

We also compared our immunomorphological results with recent single-cell transcriptomics data [14].

Although previous studies have described certain aspects of human adrenal medulla development, including the presence of large and small cell types [15] and stereological parameters [16], a systematic and detailed morphological characterization of its development, particularly regarding cytoskeletal protein dynamics, remains incomplete. In particular, the fate of vimentin—a key regulator of cell shape and migration—has not been systematically studied in developing human chromaffin cells. Here, we provide the first immunohistochemical characterization of vimentin expression in the developing human adrenal medulla, with a specific focus on the large cell (LC) population. By combining quantitative immunofluorescence with a re-analysis of publicly available single-cell transcriptomic data, we aim to bridge the gap between transcriptomic and protein-level knowledge and to provide a basis for understanding cytoskeletal remodeling during adrenal medulla maturation.

## 2. Materials and Methods

### 2.1. Autopsy Material

This study was performed on autopsy adrenal gland samples from the collection of the Laboratory of Nervous System Development, the Avtsyn Research Institute of Human Morphology of the Federal State Budgetary Scientific Institution “Petrovsky National Research Centre of Surgery”, using tissue from 33 embryos, fetuses and newborns aged from 8–9 gws (gestational weeks) to 11 days postnatally. The age was established based on clinical data. For all available clinical information, see the Supplementary Material, Table S1. In two cases, the maternal conditions included type 1 diabetes (case No. 30) and gestational diabetes (case No. 33). In all other cases, pathology of endocrine organs was absent.

In some cases, 2 adrenal glands were available; in others, we had only 1 adrenal gland; and in some cases, we had only pieces of adrenal glands. Tissue specimen collection and handling were performed in agreement with Russian legislation and the Declaration of Helsinki. Consent for use of adrenal tissue for research was obtained from a legal representative of each subject involved in this study. All protocols were approved by the Local Ethics Committee of the Federal State Budgetary Scientific Institution, the “Petrovsky National Research Centre of Surgery” (protocol No. 11; 20 December 2024).

### 2.2. Fixation and Processing

Samples of the adrenal glands were fixed in 10% buffered formalin (pH 7.0–7.4; BioVitrum, Saint Petersburg, Russia). Then, the samples were dehydrated by standard tissue protocol with IsoPrep (BioVitrum, St. Petersburg, Russia) and embedded in Histomix (BioVitrum, St. Petersburg, Russia). Serial sections (thickness: 5 μm) were taken using a Leica RM2245 microtome (Leica Biosystems, Nussloch, Germany) and an Epredia HM 325 Manual Microtome (Epredia, Kalamazoo, MI, USA). Every 20th section was deparaffinized and routinely stained with hematoxylin and eosin (H&E). The sections were examined under Leica DM2500 and Leica DM2000 light microscopes (Leica Microsystems, Wetzlar, Germany).

### 2.3. Immunofluorescence

To analyze co-localization of tyrosine hydroxylase (TH) in the LCs of the adrenal medulla with vimentin (Vim), double immunofluorescence (IF) with primary antibodies for Vim (Vimentin Monoclonal Antibody (SP20) (MA5-14564), Lot # SJ2456979A rabbit, ThermoFisher Scientific, 3747 N. Meridian Road, Rockford, IL, USA) and TH (TH (F11) sc-25269, Lot # K1104 mouse monoclonal IgG 2a, Santa Cruz Biotechnology, 10410 Finnell St., Dallas, TX, USA) was applied. For IF labeling, sections were deparaffinized and rehydrated, and antigen retrieval was performed by microwaving sections in 10 mM citric acid buffer (pH 6.0) for 10 min. Then, sections were incubated with a blocking solution consisting of 5% normal goat serum (Jackson ImmunoResearch Laboratories Inc., West Grove, PA, USA) in Tris-buffered saline plus 0.1% Tween 20 (TBST; Thermo Fisher Scientific Inc., Fremont, CA, USA) for 30 min at room temperature. Primary antibodies were diluted in 5% normal goat serum in TBST and incubated at 4 °C for 12 h. Optimal dilutions of primary and secondary antibodies were determined using a titrating assay on several adrenal sections, balancing intense staining with minimal background. For primary antibodies, the optimal dilutions were 1:500 for TH and 1:300 for Vim. Secondary antibodies were diluted in TBST and incubated at room temperature for 2 h. The following secondary antibodies and dilutions were used: iFluor 488 Conjugated Goat Anti-Mouse IgG Goat Polyclonal HUABIO (HA1125-100) (1:500) and iFluor 594 Conjugated Goat Anti-Rabbit IgG Goat Polyclonal HUABIO (H (A1122-100) (1:300) (HUABIO, 300 Tradecenter Drive, Suite 1710, Woburn, MA, USA).

Sections were covered using an EverBrite™ mounting medium with DAPI (Biotium, Fremont, CA, USA).

Negative controls were performed by replacing the primary antibodies with 1% normal goat serum in TBST.

### 2.4. Morphometry

Preparations were analyzed using an ADF U300 microscope equipped with a fluorescent block, digital microscopy camera Ultra09, and ADF Image capture software (version x64, 4.11.21522.20221011) (ADF Microscopes, Ningbo, Nanjing, China). All measurements were performed using the ADF Image capture software.

All the material was divided into 3 age groups according to our previously published data [1]: group 1 (cases No. 1–10: 8–14 gws), group 2 (cases No. 11–23: 16–21 gws), and group 3 (cases No. 24–33: 22 gws-11 days postnatally).

TH positivity indicated LCs and SCs of the AM. For our aim, only LCs were counted in the whole section of 1 or both adrenal glands (depending on availability of material in each case). Then, the number of Vim+ cells among TH+ LCs was counted (the co-localized cells).

### 2.5. Secondary Analysis of Published Single-Cell RNA-Sequencing Data

Publicly available single-cell RNA-sequencing data from developing human adrenal glands generated by Jansky S. et al. [14] were used for a targeted secondary analysis of Vim expression. The processed dataset and associated cell metadata were obtained from the repository mentioned in the original article.

Seurat 5.5 [17] was used in the R 4.6 environment for assessment of the dataset [18].

The processed Seurat object was imported into R version 4.6.0 using Seurat version 5.5. The processed count matrix, cell metadata, original UMAP coordinates, and published cell-state annotations contained in the downloaded object were retained. Read alignment, cell calling, quality control filtering, dataset integration, dimensionality reduction, clustering, and cell-type annotation were not repeated. Thus, the present work represents a targeted secondary analysis rather than a complete reprocessing of the original sequencing data.

Cells assigned to the three chromaffin lineage states labeled in the downloaded object as connecting chromaffin cells, chromaffin cells, and late chromaffin cells were selected for analysis. The resulting analytical dataset contained 3808 cells from 17 specimens: 1499 connecting chromaffin cells, 1346 chromaffin cells, and 963 late chromaffin cells. No additional filtering or reclustering of these cells was performed.

Vim expression was extracted from the RNA assay. A cell was classified as “Vim-detected” (Vim+) when its Vim value in the log-normalized data layer was greater than zero. Under the applied normalization, this criterion identifies cells with a nonzero Vim count. Because zero values in single-cell RNA-sequencing data may also reflect incomplete transcript detection, the term “Vim-detected” is used rather than implying confirmed biological absence in cells with a zero value.

For every specimen and cell-state category, we calculated the total number of cells, number of Vim-detected cells, and proportion of Vim-detected cells. For descriptive quantification of expression abundance, raw counts were normalized separately for each cell by the total number of counts in that cell and multiplied by 106, producing counts per million (CPM). The mean Vim CPM was calculated among Vim-detected cells within each specimen and cell-state category. Specimen–cell-state combinations containing no cells were reported as unavailable and did not contribute to proportion estimates or statistical models.

UMAP visualizations were generated using the coordinates stored in the published Seurat object. Cells were colored according to the retained cell-state annotations, and Vim expression was displayed using values from the log-normalized RNA data layer.

For our analysis, we chose the dataset from the article by Jansky S. et al. [14] because it contained data on later fetuses aged 7–17 pcws (post-conception weeks), unlike the dataset from the article by Kameneva P. and others [19], which was limited to fetuses aged 14 pcws.

However, data on later ages (after 17 pcws) are not present in the database of Jansky et al., yet at these very ages, significant changes in the adrenal glands take place. The age of 17 post-conception weeks (pcws) corresponds to 19 gestational weeks (gws). From this point onward, ages are expressed in gws to avoid confusion between the two conventions.

### 2.6. Statistics

#### 2.6.1. Software and Methods

Each fetus was considered one biological replicate, and all statistical analyses were performed at the specimen level. No technical replicates were included. For morphometric analysis, the largest representative histological section from each fetus was scanned in its entirety and analyzed. For the single-cell RNA sequencing analysis, biological replicates corresponded to the individual specimens included in the publicly available dataset from the article of Jansky S. et al. [14], whereas individual cells were treated as observations nested within specimens. Accordingly, specimen identity was incorporated as a random effect in the mixed-effects logistic regression model to account for within-specimen correlation. No formal sample size calculation was performed because the number of specimens was determined by the availability of suitable fetal material meeting the inclusion criteria.

StataNow/BE19.5. was used for statistical analysis (StataCorp, College Station, TX, USA). Generalized linear models were fitted using the “glm” procedure with a binomial family and logit link, whereas mixed-effects analyses were performed using the “melogit” procedure.

No formal correction for multiple comparisons was applied because the reported *p*-values correspond to predefined regression coefficients from a single generalized linear model (or mixed-effects generalized linear model), rather than to multiple post hoc pairwise tests.

#### 2.6.2. Method for Morphological Data

To assess age-related differences in vimentin expression among LCs, the number of Vim+ LCs was analyzed relative to the total number of LCs for each subject. The outcome variable represented grouped binomial data, defined as the number of vimentin-positive cells out of the total number of analyzed cells for each specimen. Because the total number of analyzed cells varied substantially among specimens, proportions estimated from different specimens had different levels of precision. Statistical methods based solely on observed proportions (ANOVA or Kruskal–Wallis) would treat these observations as equally precise and would ignore the corresponding denominators. Therefore, generalized linear models with a binomial distribution and logit link were used, allowing both the number of positive cells and the total number of analyzed cells to be incorporated directly into the analysis.

Age group was included as a categorical predictor variable. Due to substantial overdispersion in the binomial model, standard errors were corrected using Pearson χ^2^ scaling. Statistical analyses were performed in Stata (StataNow/BE19.5, College Station, TX, USA) using generalized linear modeling procedures. A *p*-value of < 0.05 was considered statistically significant.

#### 2.6.3. Method for Single-Cell Data

In the single-cell data, we did not compare the number of Vim+ cells between ages for two reasons. First, it is impossible to directly compare cell clusters detected by molecular methods with morphologically defined chromaffin cell types. Second, some cell clusters detected by single-cell analysis were absent at certain developmental stages. Therefore, we compared these clusters based on the number of Vim+ cells within them.

Since the molecular data consisted of repeated measurements obtained from three chromaffin cell clusters (CCC, CC, and LCC) within the same specimen, observations originating from the same specimen were not statistically independent. In addition, the total number of analyzed cells differed substantially among clusters and specimens, resulting in different levels of precision of the estimated proportions. Therefore, mixed-effects logistic regression with a binomial distribution and logit link was used. This approach simultaneously accounts for the grouped binomial nature of the data and the within-specimen correlation by including specimen as a random effect.

For each cluster within each specimen, the number of Vim+ cells was analyzed relative to the total number of cells in the corresponding cluster. As the outcome represented binomial count data, a mixed-effects logistic regression model with a binomial distribution and logit link function was applied. Cell cluster identity was included as a fixed effect, whereas specimen identity was included as a random intercept to account for clustering of observations within individual cases.

Model significance was evaluated using Wald χ^2^ statistics. Effect sizes were expressed as odds ratios (ORs) with corresponding 95% confidence intervals (CIs). The necessity of including a random effect was assessed using a likelihood ratio test comparing the mixed-effects model with a conventional logistic regression model. A *p*-value of < 0.05 was considered statistically significant.

## 3. Results

### 3.1. General Description of the Developing Adrenal Gland

**Figure 1:**
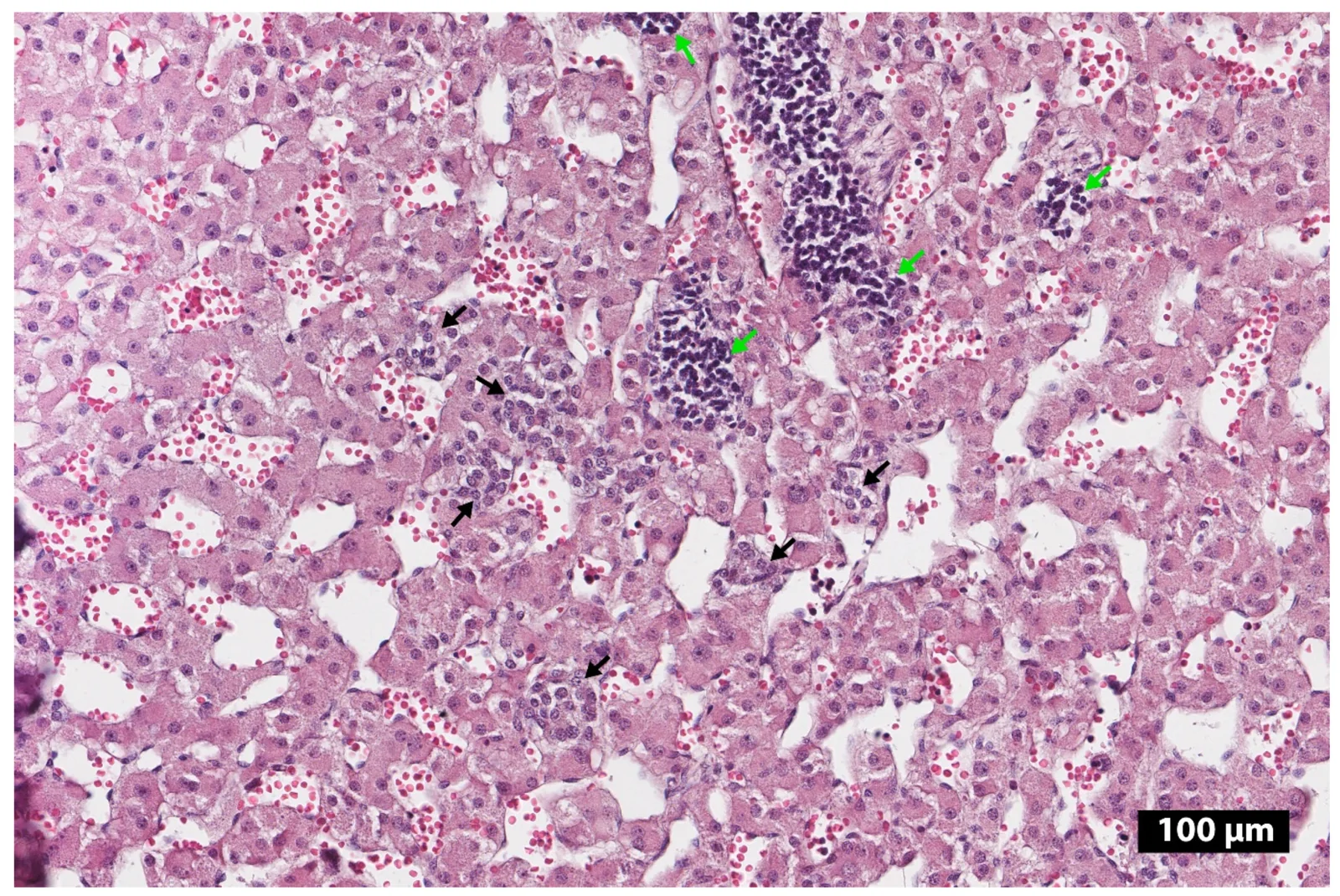
Demonstration of adrenal gland organization. Fetal cortex cells are large, with prominent eosinophilic cytoplasm. Among the cells of the fetal cortex, clusters of LCs (black arrows) and SCs (green arrows) are present. LCs are large cells with large clear nuclei and eosinophilic cytoplasm. SCs are smaller, with small hyperchromatic nuclei.

In all specimens, the adrenal glands stained with hematoxylin and eosin consisted of the cortex and the AM, which was represented by the so-called LCs and SCs (Figure 1) described in detail in our previous work [1]. Some of the cases (8–23 gws) have already been described in that work (Nos. 1–7, 11, 16, 21–23, 25, 28, and 31). At early stages (8–12 gws), we previously described that LCs migrate from the OZs to the adrenal anlagen. Newly investigated cases aged 12–23 gws did not demonstrate any significant differences in their histological appearance compared with the previously investigated cases. The new cases aged 23 gws and older deserve a description, as adrenals of these ages have not been described previously.

In all cases aged ≥ 23 gws, LCs were the predominant cell type in the AM, and SCs were seen only as small clusters in the middle of LC clusters or as single cells among LCs, similar to previously described cases aged 22–26 gws [1]. LCs were numerous and were arranged in clusters and fields, often along the central vein or among cortical parenchyma, usually in the center of the gland. Single clusters of LCs were sometimes present under the adrenal gland capsule and had connective tissue fibers interspersed between the cells and surrounding them. The LC fields in the center of the gland were not well defined from the cortical tissue, and no capsule was present around them.

All the cases of these ages (≥23 gws) demonstrated various degrees of sinusoid congestion in the adrenal cortex, with strong congestion in case No. 28 and hemorrhages in the fetal cortex (case No. 33). At the same time, the hemorrhages almost did not affect the adrenal medulla. In 1 case (No. 31), vascular congestion was mild to moderate, small sinusoid vessels were numerous in the fetal cortex, and numerous macrophages were present intravascularly. In all other cases, congestion was mild.

### 3.2. Vimentin Staining in Adrenal Medulla Cells: Heterogeneity and Regional Patterns

**Figure 2:**
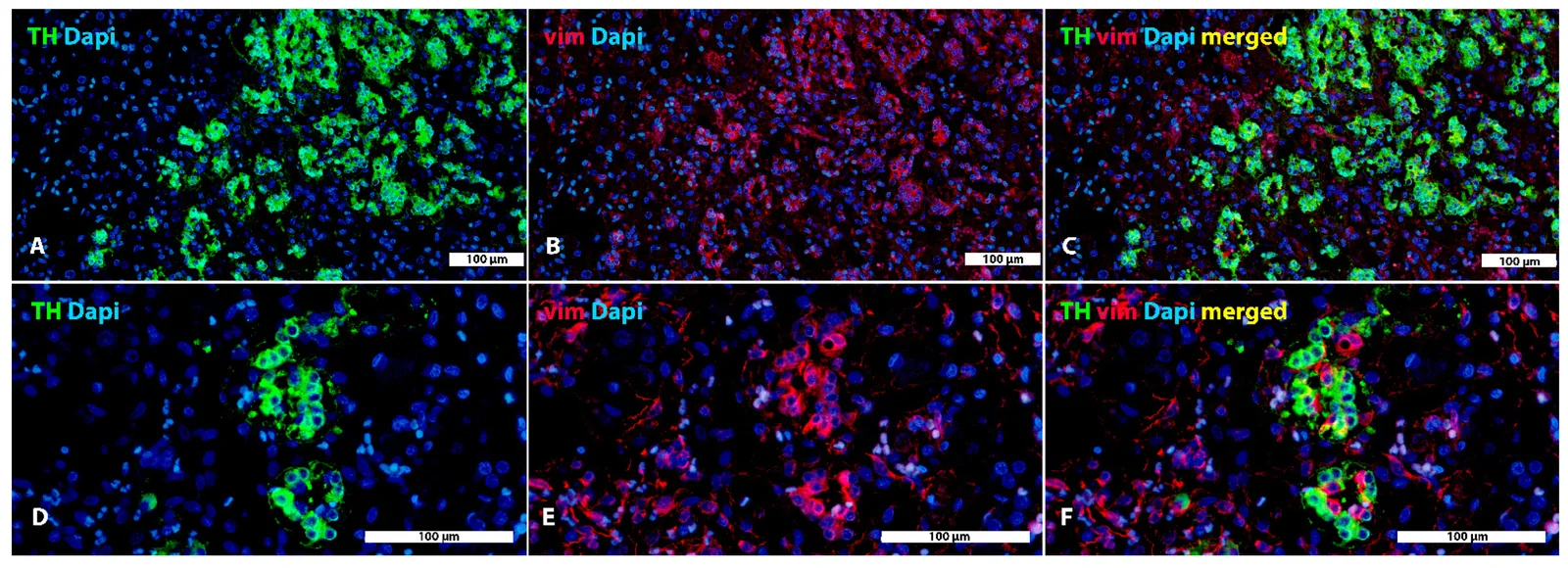
Double immunofluorescence with TH and Vim, case No. 31 (aged 32 gws + 6 days + 7 days postnatally). Nuclei are counterstained with DAPI (blue). The AM is represented by LCs, which are strongly TH+ (green) (**A**,**D**). Vimentin positivity in the LCs is moderate to strong (red) (**B**,**E**). (**C**,**F**) are merged micrographs. Scale bar = 100 μm.

Using double immunofluorescence for tyrosine hydroxylase and vimentin, we found that in all adrenal gland specimens, LCs and SCs were positive for TH, with stronger TH reactivity in the LCs. Early stages demonstrated weaker TH reactivity in the LCs than later stages. Strong TH positivity was used as a marker of the LCs (Figure 2A,D).

Vimentin was positive in many cell types in the adrenal gland: in the endothelial cells of the vessels, in the fibrous capsule of the adrenal gland, in the Schwann cells along the nerves and among the clusters of SCs and LCs. A weak reaction was also seen in the adrenal cortical cells. Vimentin staining of endothelial and Schwann cells was used as an internal positive control.

**Figure 3:**
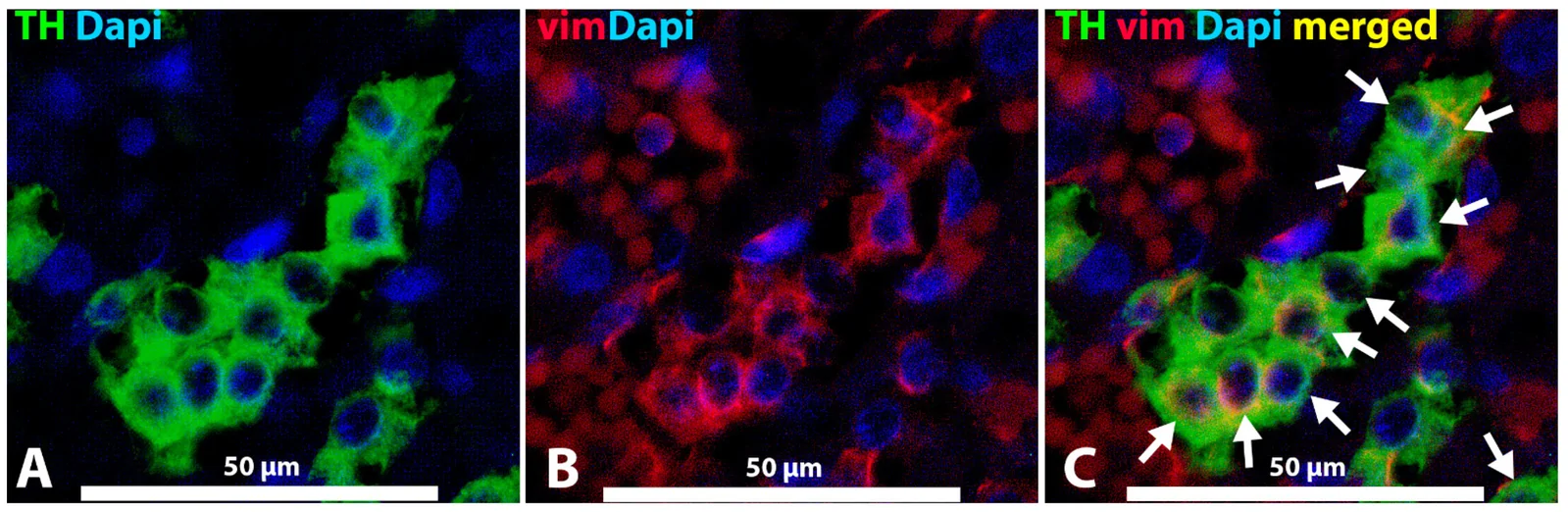
Double immunofluorescence with TH (green) and Vim (red) (case No. 20). Demonstration of co-localized TH+Vim+ LCs. Nuclei are counterstained with DAPI (blue). (**A**) LCs are TH+ (green). (**B**) Some LCs are Vim+ (red). (**C**) Merged signals; co-localized TH+Vim+ cells appear light-green/yellow/orange (arrows).

Among the LCs, vimentin reactivity ranged from weak to strong. Strong reactivity was defined when its intensity was equal to that in endothelial and Schwann cells; moderate when the reaction was pronounced but weaker than that in endothelial and Schwann cells; and weak when the reaction was barely noticeable, yet the entire cytoplasm of the cell was stained. A negative reaction was defined as the absence of complete cytoplasmic staining. Co-localization was defined when LCs were both TH+ and Vim+ (Figure 3).

At early ages (8–12 gws), vimentin positivity was weak to moderate. The latest stages, in contrast, demonstrated, on average, stronger reactivity. The strongest vimentin reactivity in the LCs was found in cases Nos. 29 and 31 (Figure 2), which had the longest postnatal periods (9 and 11 days respectively). Strong vimentin reactivity in the LCs was also present in cases Nos. 17, 26 and 30 (Supplementary Material, Table S2). In other cases, Vim reactivity was moderate, with various numbers of strongly and weakly vimentin-positive and -negative cells.

No obvious regional differences were found depending on whether the LCs of the medulla were located near the adrenal capsule or in the adrenal center, or near extra-adrenal structures such as ganglia and the OZs.

**Figure 4:**
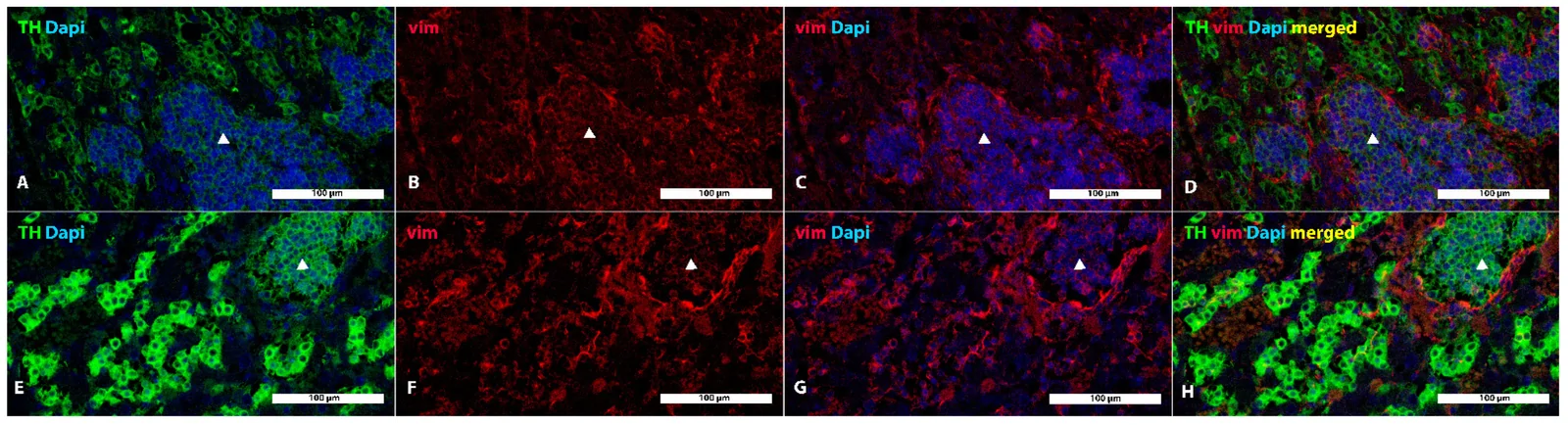
Comparison of vimentin reactivity in the LCs and SCs. Double immunofluorescence with TH (green) and Vim (red). Nuclei are counterstained with DAPI (blue). SC clusters are indicated by white triangles. LCs are located around SC clusters and are strongly TH+. (**A**–**D**) Fetus aged 19 gws (Case No. 18). SC clusters show stronger Vim reactivity than do LCs, as is most obvious on micrograph B (without Dapi channel). Strongly immunostained sustentacular cells surround SC clusters. (**E**–**H**) Fetus aged 19 gws + 4 days (Case No. 20). SC clusters show weaker immunostaining for vimentin than do LCs. Sustentacular cells around the SC cluster are strongly vimentin-positive. Scale bar = 100 μm.

Differences in vimentin positivity between the LCs and SCs were present but did not fit into a straightforward scheme. All the SC clusters were surrounded by TH-negative Vim-positive cells, which were likely sustentacular cells. As for the SCs themselves, they, in some cases, showed stronger reactivity than did the LCs, while in other cases, the opposite was true (Figure 4). Even neighboring SC clusters differed in their positivity. No relationship was found between vimentin positivity and either the shape of the SCs clusters or their location relative to LCs clusters, to each other, or to other structures. We also found no connection between the degree of vimentin positivity of the SCs clusters relative to the LCs and the age of the fetuses. However, large clusters of SCs were not observed at all stages of development, as noted above and described in more detail in our previous study [1].

**Figure 5:**
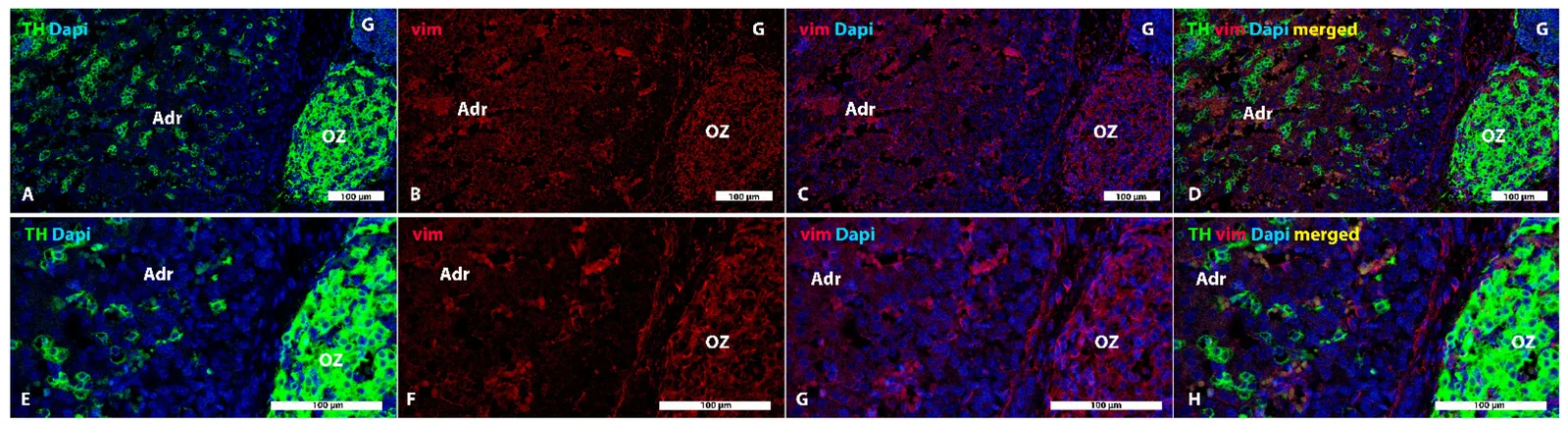
The AM and OZs: comparison of vimentin reactivity. G—ganglia; Adr—adrenal gland. Case No. 8 (aged 12–13 gws). Both the AM in the adrenal gland and the OZs are represented by LCs, which are strongly TH-positive. Vimentin positivity in the AM is equal to or even slightly weaker than that in the OZs. (**A**–**D**)—magnification ×20; (**E**–**H**)—magnification ×40 of the same area. Scale bar = 100 μm.

Although comparing the AM with the OZs was not an objective of this study, in cases where both structures were present in the same section of the adrenal gland, a vimentin reaction ranging from moderate to strong was observed in the OZs, equal to or even stronger than in the cells of the adrenal medulla that we consider to be migrating from them (Figure 5).

### 3.3. Proportion of Vimentin-Positive LCs Across Developmental Stages

The number of LCs with co-localization of TH and vimentin was counted in all cases (Table 1).

Generalized linear modeling demonstrated no statistically significant effect of age group on the proportion of TH+ Vim+ co-localized cells.

**Figure 6:**
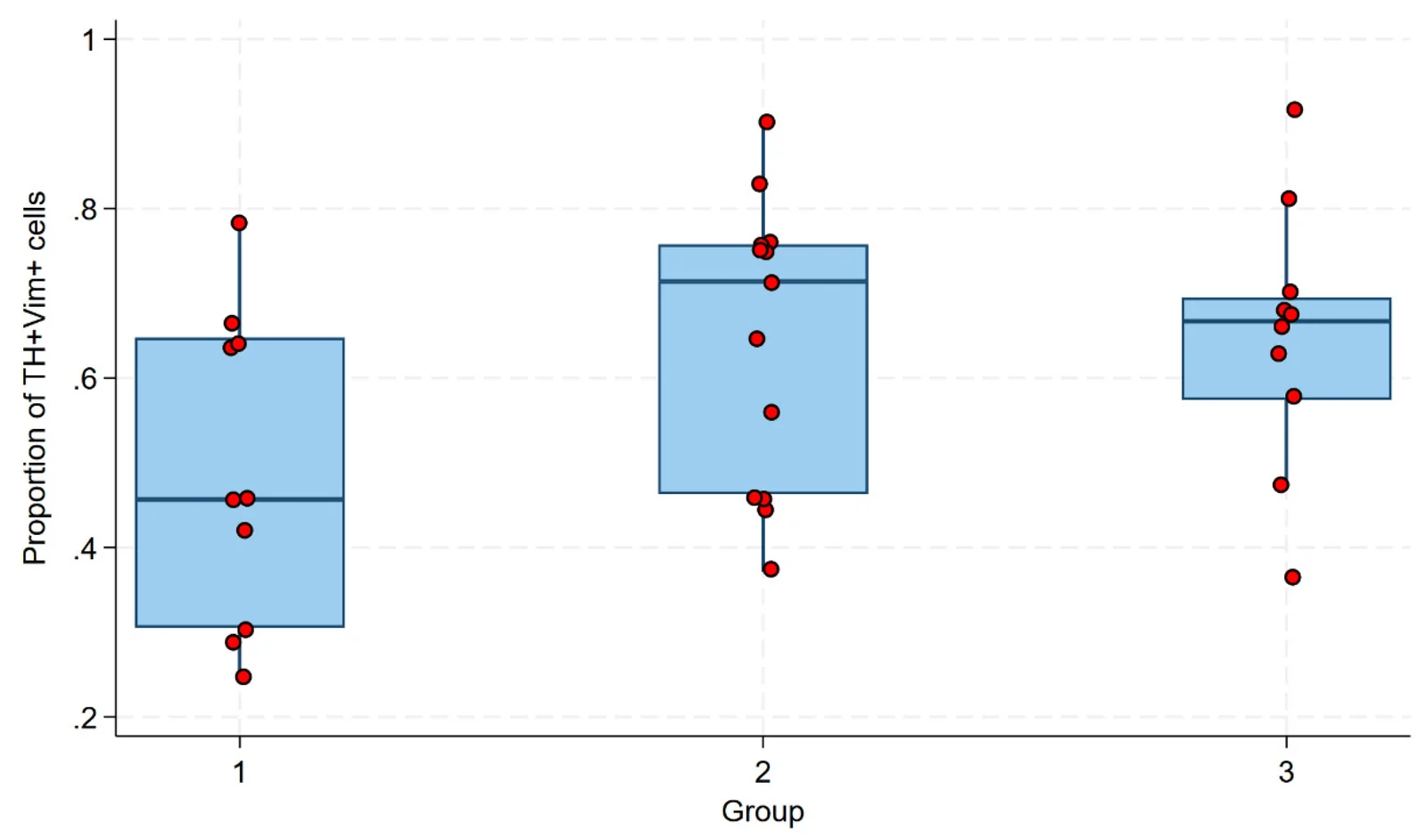
Proportion of TH+ Vim+ cells among TH-positive LCs in different age groups (1, 2, and 3). Boxplots showing the distribution of proportions within each age group with individual subject values overlaid. No statistically significant age-related differences were detected by generalized linear modeling with binomial distribution and logit link function (*p* > 0.05).

Compared with group 1, group 2 demonstrated a non-significant increase in TH+ Vim+ co-localization (β = 0.458; *p* = 0.124), while group 3 also showed a non-significant increase (β = 0.511; *p* = 0.113) (Figure 6).

The estimated odds ratios suggested a trend toward increased TH+Vim+ co-localization in older groups; however, the confidence intervals overlapped unity, and statistical significance was not reached.

Substantial overdispersion was observed in the initial binomial model, indicating greater biological variability than expected under a standard binomial distribution. Therefore, Pearson-scaled standard errors were used to obtain robust statistical inference.

### 3.4. Vimentin Expression in Single-Cell Transcriptomic Clusters

**Figure 7:**
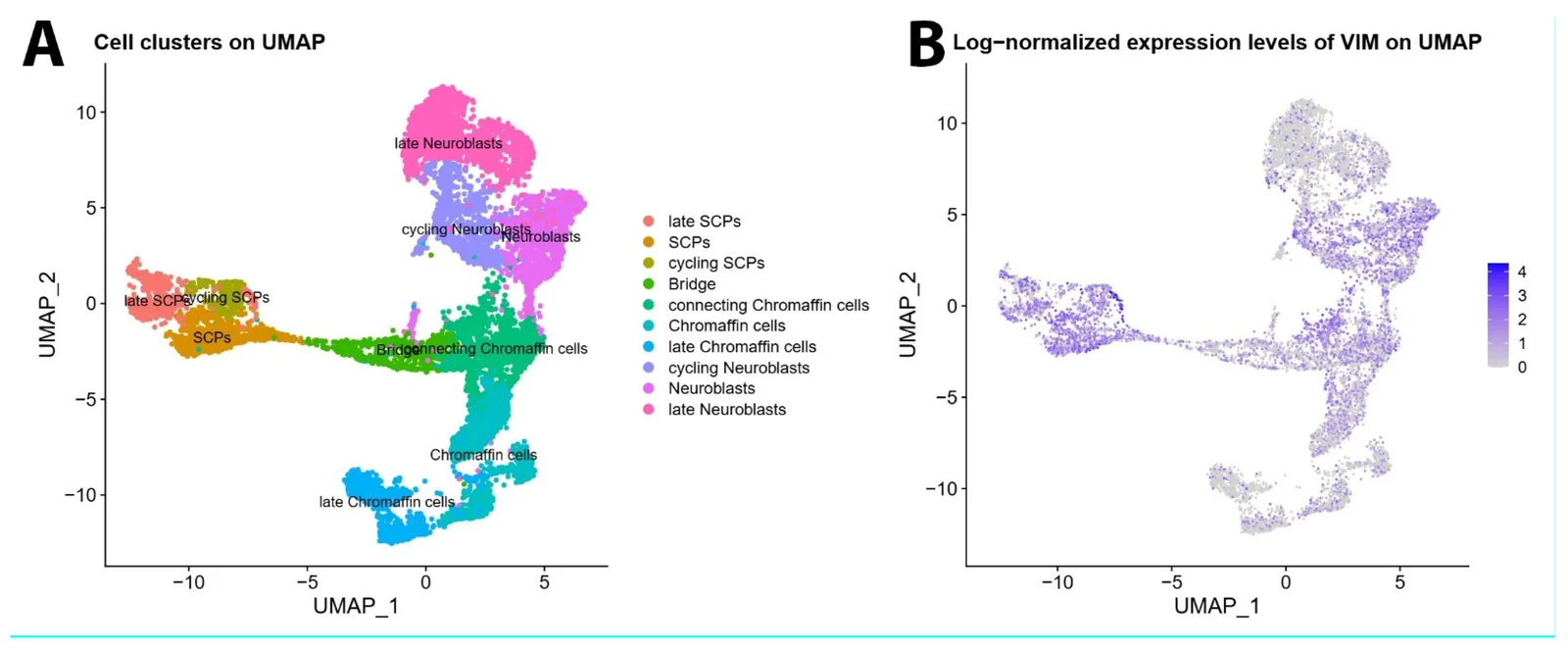
UMAP of the scRNA-seq dataset from Jansky et al. [14], re-analyzed and visualized in this study. (**A**) Cells are colored according to cluster identity (cell types as in the reference paper). SCPs—Schwann cell precursors. (**B**) Log-normalized, library-size-normalized expression levels of the target gene Vim.

Initial separation of cell clusters was as follows: clustering was similar to that described in the article (Figure 7); clusters of neuroblasts, chromaffin cells, and Schwann cell precursors were identified. Among chromaffin cells, clusters of connecting chromaffin cells, chromaffin cells and late chromaffin cells were identified.

In the clusters of connecting chromaffin cells (CCCs), chromaffin cells (CCs) and late chromaffin cells (LCCs), the total number of cells in each case, the number of Vim+ cells, the percentage of Vim+ cells relative to all cells in these clusters, and the average number of vimentin RNA reads per cell per million (CPM) were determined (Table 2). Cells were considered Vim+ (“positive”) when the log-normalized expression level of the gene in a given cell was >0.

During the analysis of the scRNA-Seq dataset of the AM during development [14], we found that mixed-effects logistic regression revealed a significant effect of cell cluster identity on vimentin positivity (Wald χ^2^ = 9.67; *p* = 0.008).

No significant difference in vimentin expression was observed between CCCs and chromaffin cells (CCs) (OR = 1.16, 95% CI 0.84–1.61, and *p* = 0.366).

In contrast, late chromaffin cells (LCCs) demonstrated significantly lower vimentin positivity compared with chromaffin cells (CCs) (OR = 0.36, 95% CI 0.18–0.72, and *p* = 0.004). Thus, the odds of vimentin expression in LCCs were approximately 64% lower than in CCs.

The variance of the random intercept indicated substantial between-specimen variability. Consistently, the mixed-effects model provided a significantly better fit than a conventional logistic regression model (likelihood-ratio test: chibar^2^ = 124.80; *p* < 0.001), confirming the importance of accounting for within-specimen clustering.

These findings indicate that the proportion of Vim+ cells remains comparable between CCCs and CCs but decreases significantly in LCCs.

## 4. Discussion

At first glance, our double immunofluorescence data and the single-cell transcriptomic data (scRNA-seq) from other authors appear to show discrepancies. Immunofluorescence shows that the ratio of Vim+ cells does not change significantly with age, and the staining intensity even appears to be higher in later fetuses, being the strongest in postnatal samples. In contrast, analysis of the single-cell RNA-seq dataset reveals a decrease in the ratio of Vim+ cells in the subpopulation of LCCs, whose number increases with age.

These differences can be largely explained by fundamental methodological differences between the two approaches. The scRNA-seq dataset quantifies mRNA levels, whereas immunofluorescence is not a quantitative method and assesses protein rather than transcript. However, the most important factor likely is the exceptional stability of vimentin protein. Once formed, vimentin filaments are remarkably stable and can persist for long periods without fragmentation (tens of hours) [20], even when transcription declines—in stark contrast to vimentin mRNA, which has a relatively short half-life (approximately 6 h in chicken cell culture), whereas vimentin protein has a half-life of 31–32 h (32 h in mouse fibroblasts and 31 h in chicken cell culture) [21]. Consequently, the presence of vimentin protein in cells (detected by immunofluorescence) may outlast its corresponding mRNA (detected by scRNA-seq). Additional factors, such as a temporal delay between transcription and filament incorporation, as well as possible transcriptional changes induced by tissue dissociation procedures, may further contribute to the observed discrepancies. Moreover, technical limitations inherent to scRNA-seq may also contribute to the observed discrepancies. Dropout events, sequencing depth, batch effects, and dissociation-induced transcriptional stress can further affect gene detection and cell representation [22]. Thus, the lower proportion of Vim+ cells in LCC clusters may partly reflect technical artifacts rather than a genuine biological decrease. Therefore, a direct comparison of these heterogeneous data is not appropriate, and the results should be viewed as complementary. Furthermore, scRNA-seq has not been applied to samples older than 19 gws, which also limits direct comparisons with our data.

Next, our immunofluorescent data show that vimentin is present in a portion of chromaffin cells, and this fact raises two interconnected questions: what functions does vimentin play in chromaffin cells, and why is only a portion of chromaffin cells (LCs) vimentin-positive?

If at the early stages, the hypothetical role of vimentin is quite clear, as the LCs are migrating to the adrenal anlage, its role in more differentiated chromaffin cells at later ages remains more enigmatic.

We propose that the extensive remodeling of the adrenal gland, which begins prenatally and continues for up to a year after birth, requires chromaffin cells to undergo substantial shape changes. Vimentin may be a key regulator of this process. Such a process involving vimentin was demonstrated, for example, in developing neurons. In the work of Kumano et al. [5], a positive vimentin reaction was shown not only in chicken migrating neural crest cells but also in a portion of the so-called large round cells corresponding to neurons of the cervical dorsal root ganglia at stage 36. The authors proposed the following scheme of intermediate filament change in developing neurons: vimentin → nestin → neurofilament protein → vimentin + neurofilament protein → neurofilament protein. Interestingly, the authors attribute this vimentin reappearance to a change in the morphology of neurons from bipolar to pseudounipolar that occurs during this very stage. Moreover, vimentin is essential for neuritogenesis in cell cultures and also contributes to axon elongation [23]. Transient appearance of vimentin also occurs in some other tissues and is also claimed to be connected with cell shape changes [24].

Besides this, vimentin may play a role in catecholamine secretion, at least in cell cultures [11]. However, the exact mechanism and whether it occurs in vivo remain unknown. The known functional role of vimentin has greatly expanded beyond that of a protein simply maintaining cell shape; it is now recognized as a regulator of many biological processes, including the regulation of transcription factors and genomic DNA [25].

Whether vimentin positivity correlates with other forms of chromaffin cell heterogeneity remains unknown. For example, vimentin could potentially mark either the primary or the secondary LC population.

It is interesting that such heterogeneity is also present in postnatal life. For instance, in the adult human AM, half of the chromaffin cells were shown to be vimentin-positive [13]. It could be that this heterogeneity, with respect to vimentin, correlates with some other form of heterogeneity. In a recent study on mice [26], five clusters of chromaffin cells were distinguished in adult mice using single-cell RNA sequencing. Vimentin was not shown to be a marker of any of these clusters, but given the stability and long half-life of vimentin, single-cell data may not reflect the protein content of the cells.

## 5. Limitations of This Study and Conclusions

Several limitations should be considered. First, the sample size was relatively small, and the autopsy material was inherently variable in tissue preservation and fixation, which may affect immunostaining quality. Second, vimentin intensity was assessed semi-quantitatively relative to internal controls (endothelial and Schwann cells) rather than by absolute measurement. Third, the scRNA-seq data used for comparison were obtained from a published dataset, limiting direct comparability. Fourth, morphologically defined LCs do not directly correspond to molecularly defined CCC, CC, and LCC clusters. Fifth, the proposed roles of vimentin remain speculative.

Although the persistence of vimentin in LCs raises intriguing questions about its possible functions—such as involvement in cytoskeletal remodeling, cell migration, differentiation, or neuroendocrine secretion—our study was not designed to address these mechanistically. The available literature on vimentin functions in chromaffin and neural crest-derived cells remains limited and fragmentary, and direct evidence for specific roles in developing human adrenal medulla is currently lacking.

In summary, this study is the first to systematically characterize vimentin expression in developing human adrenal medulla LCs. We show that vimentin protein persists throughout development despite declining transcript levels in late chromaffin cells, reflecting both the stability of vimentin filaments and chromaffin cell heterogeneity. These findings suggest a role for vimentin in cytoskeletal remodeling during adrenal medulla maturation, although its precise functions remain to be determined. Future functional studies are needed to define the roles of vimentin in chromaffin cell biology.

## Figures and Tables

**Table 1:** Numbers of TH+Vim+ co-localized cells and total TH+ LCs per case, and the ratio of TH+Vim+ cells to TH+ LCs.

Case No.	Age	TH+ LCs	Number of TH+Vim+ Cells	The Ratio of TH+Vim+ Cells to TH+LCs	Age Group
1	8.5	184	117	0.63587	1
2	8.5	442	135	0.30543	1
3	9	167	70	0.419162	1
4	11	258	167	0.647287	1
5	11.5	460	135	0.293478	1
6	11.5	327	82	0.250765	1
7	12	1846	1430	0.774648	1
8	12.5	1921	1278	0.665279	1
9	12.9	4227	1945	0.460137	1
10	14.50	2327	1054	0.452944	1
11	16	66	50	0.757576	2
12	16.00	6952	3094	0.445052	2
13	16.00	648	364	0.561728	2
14	16.50	1722	1289	0.748548	2
15	16.50	730	338	0.463014	2
16	17	652	498	0.763804	2
17	17.5	2750	2471	0.898545	2
18	19	4269	3189	0.747013	2
19	19	281	183	0.651246	2
20	19.6	3311	2364	0.713984	2
21	20.50	616	229	0.371753	2
22	21	848	390	0.459906	2
23	21.5	637	528	0.828885	2
24	22.5	423	266	0.628842	3
25	22.5	2221	1506	0.678073	3
26	23.5	1139	1045	0.917471	3
27	24	524	301	0.574427	3
28	25.6	507	409	0.806706	3
29	28.7	4465	2974	0.666069	3
30	31.4	400	190	0.475	3
31	33.9	3493	2427	0.694818	3
32	38	807	539	0.667906	3
33	39.5	2026	753	0.371668	3

**Table 2:** Absolute number and percentage of Vim+ cells among three clusters of chromaffin cells, CCC, CC, and LCC, and the average number of vimentin RNA reads per cell, normalized to counts per million (CPM) (mean Vim CPM).

Case	Age, gws	CCC				CC				LCC			
		Total	Vim+	Vim+ %	Mean Vim CPM	Total	Vim+	Vim+ %	Mean Vim CPM	Total	Vim+	Vim+ %	Mean Vim CPM
12614	9 gw	378	294	77.78	724.74	6	6	100.00	451.16	0	0	—	—
13810	9 gw	7	2	28.57	706.16	37	12	32.43	286.99	1	1	100.00	960.61
14607	9 gw	642	299	46.57	511.09	6	3	50.00	276.49	0	0	—	—
13363	10 gw	53	17	32.08	394.42	577	248	42.98	294.31	1	1	100.00	249.07
13567	10 gw	10	2	20.00	228.86	26	3	11.54	315.22	0	0	—	—
14933	10 gw	398	235	59.05	406.98	77	38	49.35	466.50	0	0	—	—
15084	10 gw	3	1	33.33	692.52	338	112	33.14	377.47	8	4	50.00	459.22
15161	10 gw	5	2	40.00	316.79	272	100	36.76	244.77	0	0	—	—
13667	13 gw	0	0	—	—	0	0	—	—	166	16	9.64	738.97
13952	13 gw	0	0	—	—	0	0	—	—	109	8	7.34	763.88
14164	13 gw	0	0	—	—	3	0	—	—	341	67	19.65	404.44
14490	16 gw	2	2	100.00	499.32	0	0	—	—	50	17	34.00	549.45
14742	16 gw	0	0	—	—	0	0	—	—	32	4	12.50	1086.82
14766	16 gw	0	0	—	—	3	1	33.33	432.71	124	16	12.90	743.88
14627	19 gw	0	0	—	—	0	0	—	—	58	4	6.90	681.15
14707	19 gw	0	0	—	—	1	1	100.00	239.64	31	4	12.90	235.03
14773	19 gw	1	0	—	—	0	0	—	—	42	4	9.52	831.85

## Data Availability

The data presented in this study are contained within this article and Supplementary Material (Tables S1 and S2).

## Supplementary Materials

The following supporting information can be downloaded at https://www.mdpi.com/article/10.3390/life16081295/s1. Table S1: Clinical information; Table S2: Vimentin immunoreactivity intensity in Vim+ LCs.

## Abbreviations

The following abbreviations are used in this manuscript:

AM	Adrenal medulla
OZs	Organs of Zuckerkandl
LCs	Large cells
SCs	Small cells
TH	Tyrosine hydroxylase
Vim	Vimentin
pcws	Post-conception weeks
gws	Gestational weeks
CCCs	Connecting chromaffin cells
CCs	Chromaffin cells
LCCs	Late chromaffin cells

## References

[1] Otlyga E., Otlyga D., Junemann O., Krivova Y., Proshchina A., Kharlamova A., Gulimova V.I., Sonin G., Saveliev S. (2025). Developmental Parallels Between the Human Organs of Zuckerkandl and Adrenal Medulla. Life.

[2] Lewis R.W., Pappenheimer A.M. (1916). A Study of the Involutional Changes Which Occur in the Adrenal Cortex during Infancy. J. Med. Res..

[3] Benner M.C. (1940). Studies on the Involution of the Fetal Cortex of the Adrenal Glands. Am. J. Pathol..

[4] Arai S., Shimizu K. (1970). Transformation of the Permanent Zone of the Adrenal Cortex in Mature and Premature Infants. Tohoku J. Exp. Med..

[5] Kumano I., Iwatsuki H., Sasaki K. (2002). Ganglion Cell Differentiation and Intermediate Filaments in the Cervical Dorsal Root Ganglion of the Chick Embryo. Kawasaki Med. J..

[6] Usman S., Waseem N.H., Nguyen T.K.N., Mohsin S., Jamal A., Teh M.T., Waseem A. (2021). Vimentin Is at the Heart of Epithelial Mesenchymal Transition (Emt) Mediated Metastasis. Cancers.

[7] Mendez M.G., Kojima S., Goldman R.D. (2010). Vimentin Induces Changes in Cell Shape, Motility, and Adhesion during the Epithelial to Mesenchymal Transition. FASEB J..

[8] Duband J.L. (2010). Diversity in the Molecular and Cellular Strategies of Epithelium-to- Mesenchyme Transitions: Insights from the Neural Crest. Cell Adh. Migr..

[9] Kameda Y. (1996). Immunoelectron Microscopic Localization of Vimentin in Sustentacular Cells of the Carotid Body and the Adrenal Medulla of Guinea Pigs. J. Histochem. Cytochem..

[10] Suzuki T., Kachi T. (1995). Immunohistochemical Studies on Supporting Cells in the Adrenal Medulla and Pineal Gland of Adult Rat, Especially on S-100 Protein, Glial Fibrillary Acidic Protein and Vimentin. Kaibogaku Zasshi.

[11] Quintanar J.L. (2000). Vimentin in Cultured Chromaffin Cells: An Immunofluorescent, Biochemical and Functional Study. Cell. Physiol. Biochem..

[12] Bertelli E., Regoli M., Lucattelli M., Bastianini A., Fonzi L. (2002). Nestin Expression in Rat Adrenal Gland. Histochem. Cell Biol..

[13] Henzen-Logmans S.C., Stel H.V., van Muijen G.N.P., Mullink H., Meijer C.J.L.M. (1988). Expression of Intermediate Filament Proteins in Adrenal Cortex and Related Tumours. Histopathology.

[14] Jansky S., Sharma A.K., Körber V., Quintero A., Toprak U.H., Wecht E.M., Gartlgruber M., Greco A., Chomsky E., Grünewald T.G.P. (2021). Single-Cell Transcriptomic Analyses Provide Insights into the Developmental Origins of Neuroblastoma. Nat. Genet..

[15] Molenaar W.M., Lee V.M.-Y., Trojanowski J.Q. (1990). Early Fetal Acquisition of the Chromaffin and Neuronal Immunophenotype by Human Adrenal Medullary Cells. An Immunohistological Study Using Monoclonal Antibodies to Chromogranin A, Synaptophysin, Tyrosine Hydroxylase, and Neuronal Cytoskeletal Proteins. Exp. Neurol..

[16] Bocian-Sobkowska J., Wozniak W., Malendowicz L.K., Ginda W. (1996). Stereology of Human Fetal Adrenal Medulla. Histol. Histopathol..

[17] Hao Y., Stuart T., Kowalski M.H., Choudhary S., Hoffman P., Hartman A., Srivastava A., Molla G., Madad S., Fernandez-Granda C. (2024). Dictionary Learning for Integrative, Multimodal and Scalable Single-Cell Analysis. Nat. Biotechnol..

[18] R Core Team (2026). R: A Language and Environment for Statistical Computing.

[19] Kameneva P., Artemov A.V., Kastriti M.E., Faure L., Olsen T.K., Otte J., Erickson A., Semsch B., Andersson E.R., Ratz M. (2021). Single-Cell Transcriptomics of Human Embryos Identifies Multiple Sympathoblast Lineages with Potential Implications for Neuroblastoma Origin. Nat. Genet..

[20] Tran Q.D., Lenz M., Lamour G.I., Paty L.I., Varela-Salgado M.I., Campillo C., Wioland H.I., Jegou A.I., Romet-Lemonne G.I., Leduc C. (2025). Continuous Self-Repair Protects Vimentin Intermediate Filaments from Fragmentation. Proc. Natl. Acad. Sci. USA.

[21] Coleman T.R., Lazarides E. (1992). Continuous Growth of Vimentin Filaments in Mouse Fibroblasts. J. Cell Sci..

[22] Adil A., Kumar V., Jan A.T., Asger M. (2021). Single-Cell Transcriptomics: Current Methods and Challenges in Data Acquisition and Analysis. Front. Neurosci..

[23] Dubey M., Hoda S., Chan W.K.H., Pimenta A., Ortiz D.D., Shea T.B. (2004). Reexpression of Vimentin in Differentiated Neuroblastoma Cells Enhances Elongation of Axonal Neurites. J. Neurosci. Res..

[24] Iwatsuki H., Sasaki K., Suda M., Itano C. (1999). Vimentin Intermediate Filament Protein as Differentiation Marker of Optic Vesicle Epithelium in the Chick Embryo. Acta Histochem..

[25] Ivaska J., Pallari H.M., Nevo J., Eriksson J.E. (2007). Novel Functions of Vimentin in Cell Adhesion, Migration, and Signaling. Exp. Cell Res..

[26] Santambrogio A., Kemkem Y., Willis T.L., Berger I., Kastriti M.E., Faure L., Russell J.P., Lodge E.J., Yianni V., Kövér B. (2025). SOX2+ Sustentacular Cells Are Stem Cells of the Postnatal Adrenal Medulla. Nat. Commun..

