# Supplementary material for: Vimentin in Developing Human Adrenal Medulla Cells
Source: Life (Basel). 2026 Aug 6;16(8):1295. doi: 10.3390/life16081295 (PMC13514855; doi:10.3390/life16081295)
Supplement: Supplementary file 1 [file life-16-01295-s001.zip › life-4414599-supplementary.pdf]

Table S1. Clinical information.

Case No	Age, g.w.	Clinical data
1	8-9	Dichorionic diamniotic twins. Uterine scar after 2 cesarean sections, retrochorionic hematoma.
2	8-9	
3	9	Chorion ingrowth into the uterine scar clinically. During the histological investigation of embryonic material, huge microcalcifications were found in the brain, spinal cord, and ribs of unknown nature. Pathologies in other organs were not found.
4	11	Deficiency of the uterine scar after 3 cesarean sections, premature detachment of the ovum
5	11-12	Deficiency of the uterine scar after 3 cesarean sections, detachment of the ovum
6	11-12	Incompetent uterine scar. Ultrasound signs of chorioinvasion into the scar. Destruction of the lower third of the anterior uterine wall with formation of a uterine hernia.
7	12	Cervical cancer. Tis Nx M0. Spontaneous miscarriage.
8	12-13	Body length 9,5 cm, weight less than 30 g. Congenital malformatiom of the lymphatic system - cystic hygrome of the posterior neck. Extreme immaturity. Acute intrapartum asphyxia with hemorrhages into internal organs and tissues, liquid state of the blood.
9	12 g.w. + 6 days	Weight: 30,7 g; body length: 100 cm.
10	14-15	Male sex. Intrapartum fetal hypoxia. Extreme immaturity: fetal weight 100 g, body length 15 cm. Maternal condition: multiple giant uterine leiomyomas, with symptoms of bleeding, symptoms of compression of adjacent organs, and a risk of miscarriage.
11	16	Late spontaneous miscarriage. Amputation of the cervix due to carcinoma in situ.
12	16	Cases 12-13: a late spontaneous miscarriage of a dichorionic diamniotic twin pregnancy. Isthmic-cervical insufficiency.<br>Male sex.<br>Primary disease:<br>Acute antenatal hypoxia of the second twin from a dichorionic diamniotic twin pregnancy: congestion of internal organs and tissues.<br>Extreme immaturity – 16 weeks of gestation (fetal weight 130 g, length 17 cm).<br>Maternal background conditions:<br>Isthmic-cervical insufficiency.<br>Multiple uterine leiomyomas.
13	16	Primary disease:<br>Acute antenatal hypoxia of the first twin from a dichorionic diamniotic twin pregnancy: congestion of internal organs and tissues.<br>Extreme immaturity – 16 weeks of gestation (fetal weight 140 g, length 17 cm).<br>Maternal background conditions:<br>Isthmic-cervical insufficiency.<br>Multiple uterine leiomyomas.
14	16-17	Primary disease:

		Extreme prematurity: 16 weeks and 6 days of gestation, fetal weight 130 g, length 20 cm; congestion of internal organs and tissues, liquid state of the blood.<br>Background condition:<br>Acute ascending amniotic infection with fetal response: membranitis, subchorionic intervillitis, chorioamnionitis with vasculitis; premature maturation of the villous tree; preterm premature rupture of membranes.
15	16-17	Primary disease:<br>Acute intrapartum fetal hypoxia: liquid state of blood in the cardiac chambers, congestion of internal organs and tissues. Placenta of normal weight, consistent with gestational age.<br>Background condition:<br>Extreme immaturity: 16–17 weeks of gestation, fetal weight 100 g, body length 18 cm.
16	17	Late spontaneous miscarriage
17	17-18	Primary disease:<br>Multiple congenital malformations of the fetus: cerebellar hypoplasia; large cystic lymphatic malformation (hygroma) of the posterior neck surface (2.5 cm); ventricular septal defect with dextraposition of the aorta; polycystic right kidney.<br>Extreme immaturity: 17–18 weeks of gestation, fetal weight 150 g, body length 17 cm.
18	19	Primary disease:<br>Acute ascending amniotic infection: subchorionic intervillitis, focal chronic basal deciduitis with lymphoplasmacytic infiltration; premature maturation of the villous tree. Marginal cord insertion.<br>Signs of fetal death at 19 weeks of gestation:<br>Liquid state of blood in the cardiac chambers; extreme immaturity (weight 230 g, length 21 cm); post-status of late spontaneous miscarriage.
19	19	Male sex. Body weight: 280 g, body length: 20 cm. Antepartum intrauterine hypoxia. Late induced miscarriage. Squamous cell carcinoma in the mother. Teratoma with malignant transformation of the right ovary. Early signs of acute ascending amniotic infection. Anhydramnios.
20	19 g.w. + 4 days	Primary disease:<br>Congenital malformation of the central nervous system: severe ventriculomegaly (dilatation of the cerebral ventricular system) with dysraphism and loss of cerebral hemispheric white matter.<br>Acute induced fetal hypoxia: marked congestion of internal organs, liquid blood in the cardiac chambers and great vessels.<br>Extreme immaturity (19.1 weeks of gestation, fetal weight 220 g, body length 22 cm).
21	20-21	Complete hydatidiform mole of 1 of the twins
22	21	COVID-19, systemic lupus erythematosus in mother
23	21-22	Termination of pregnancy for fetal reasons: spina bifida, Arnold-Chiari Syndrome, placental haematoma, toxic goiter (medical correction), colpitis, caused by Trichomonas vaginalis, uterine scar from 2 cesarean sections.

24	22-23	Birth weight: 460 g, birth length: 23 cm. Primary disease: Intrapartum death of the first twin from a monochorionic diamniotic twin pregnancy with signs of twin-twin transfusion syndrome (TTTS) with a weight-growth discordance of up to 26% (Twin 1: 460 g / 26 cm, Twin 2: 340 g / 23 cm). Extreme immaturity – 22.4 weeks of gestation (fetal weight 460 g, length 23 cm).<br>Background condition. Early signs of acute ascending amniotic infection – focal membranitis; second trimester placenta with signs of fetal thrombotic vasculopathy, foci of chronic basal deciduitis.
25	22-23	Severe preeclampsia
26	23-24 + 4 days 17h	Live-born, birth weight: 340 g, birth length: 28 cm<br>Primary disease:<br>Extreme immaturity: 23–24 weeks of gestation, weight 340 g, low position of the umbilical ring, canalicular stage of lung development, large amount of mesenchyme along the bronchial tree and hepatic tracts.<br>Background condition:<br>Placenta with weight at the lower limit of normal, normal blood flow, moderately expressed hemorrhages, hemorrhages into the posterior cranial fossa and under the soft meninges of the left occipital lobe, anemia of parenchymal organs.
27	24	Female sex. Body weight: 800 g, body length: 33 cm. Acute amniotic infection, antenatal fetal death.
28	25 g.w. + 4 days	Operative delivery due to severe preeclampsia, antenatal fetal death. Stillborn premature fetus.
29	28 g.w. + 3 days+ 9 days after birth	Primary diagnosis: Congenital pneumonia, unspecified.<br>Competing diagnosis: Intraventricular (nontraumatic) hemorrhage, grade 3, on the right<br>Background diagnosis: Extreme immaturity. Prematurity, 27 weeks 3 days. Extremely low birth weight<br>Complications of the primary disease: Respiratory failure of the newborn. Pneumothorax originating in the perinatal period. Neonatal sepsis caused by other bacterial agents. Septic shock. Periventricular hemorrhagic stroke, right side.<br>Concomitant diagnoses: Respiratory distress syndrome of the newborn. Cerebral ischemia
30	31 g.w. + 3 days	Female sex. Body weight: 1480 g. Body length: 39 cm. Neonatal respiratory distress syndrome. Emergency cesarean section. Reduced placental weight, utero-placental ischemia. Necrotizing enterocolitis. Maternal conditions: type I diabetes mellitus, severe preeclampsia.
31	32 g.w. + 6 days + 11 days after birth	Primary diagnosis: Congenital pneumonia.<br>Competing diagnosis: Intraventricular (nontraumatic) hemorrhage, grade 2-3<br>Complication of the primary disease: Respiratory failure of the newborn. Acute renal failure, unspecified. Pneumothorax originating in the perinatal period, left side.<br>Concomitant diagnoses: Renal dysplasia. Anemia, unspecified
32	38	Primary disease: Congenital bacterial pneumonia<br>Background condition: Newborn immaturity (gestational age 38 weeks, weight 2720 g, length 49 cm); absence of Beclard nuclei in the lower femoral epiphyses, low position of the umbilical ring; pathological immaturity of the placental villous tree.

		Complications of the primary disease: Bullous emphysema in the area of the 5th (medial) segment of the right lung, scattered atelectasis and hyaline membranes of the lungs. Spontaneous right-sided pneumothorax originating in the perinatal period. Bilateral hydrothorax, hydropericardium, ascites, edema of the retroperitoneal tissue, gallbladder wall, and testicular membranes.<br>Maternal conditions: Gestational pyelonephritis, pregnancy-induced proteinuria, asymptomatic bacteriuria. Multiple large uterine leiomyomas.<br>Lived for 22 hours.
33	39-40	Male sex. Body weight: 3000 g, body length: 50 cm. Cardiomegaly. Antenatal fetal death. Vaginal delivery. Maternal condition: maternal diabetes mellitus.

Table S2. Vimentin Immunoreactivity Intensity in Vim+ LCs. Intensities are indicated as follows: + weak, ++ moderate, +++ strong. Multiple entries indicate heterogeneity within the case, listed in descending order of prevalence.

Case No.	Vim+ intensity in LCs
1	++/+
2	++/+
3	++
4	++/+++/+
5	++/+/+++
6	+/++
7	+/++
8	++/+++
9	+/++
10	++
11	++/+
12	++/+++
13	++/+++
14	++/+++
15	++
16	++/+++
17	+++
18	++/+++
19	++/+++
20	++ (in some clusters + to +++)
21	++/+
22	++/+
23	++/+++
24	++/+++
25	++/+
26	+++
27	++/+++
28	++/+++/+

29	+++/++
30	+++/++/+
31	+++/++
32	++/+
33	++/+/+++
